# Introduction of APP induces bioenergetic deficits within an aged humanized APOE risk model of Alzheimer's disease: An FDG‐PET analysis

**DOI:** 10.1002/alz70856_107527

**Published:** 2026-01-09

**Authors:** Avnish Bhattrai, Adam C. Raikes, John W. McLean, Roberta Diaz Brinton

**Affiliations:** ^1^ University of Arizona, Tucson, AZ, USA

## Abstract

**Background:**

Late‐onset Alzheimer's disease (LOAD) affects approximately 95% of the clinical dementia population aged 65 and older, influenced by genetics, age and sex. Apolipoprotein e4 (APOE4) is the strongest genetic risk factor, in addition to other prominent risk genes such as amyloid precursor protein (APP). APOE4 confers a 15‐fold higher risk in females compared to males. Preclinical research often uses familial AD risk factor gene mouse models, which impact <5% of the clinical population, limiting clinical translatability. Herein, we investigated the impact of a single strong AD risk factor gene (APP) combined with LOAD‐specific risk factors on brain and peripheral bioenergetics.

**Method:**

Aged (23‐25 months) humanized APP/APOE (APP carrier) and hAPOE (APP non‐carrier) mice with ε3/3, ε3/4 and ε4/4 genotype underwent metabolic and body composition screening including fasting blood glucose (FBG) and ketone (FKB) measurement, EchoMRI, and 18F‐FDG‐PET. Cerebral FDG‐PET standardized uptake values (SUVR) were normalized to pons. For analysis, mice were classified as APOE4 carriers and non‐carriers, based on the presence of an ε4 allele. All data were analyzed with APP carrier x APOE carrier x sex analyses of variance followed by post‐hoc Bonferroni correction.

**Result:**

APP carriers had significantly lower SUVR compared to non‐carriers. Irrespective of genetic model, females had lower brain glucose uptake than males. APP carriers, irrespective of genotype, additionally had lower FBG than non‐carriers with a trend towards sex differences. Finally, APP carriers had higher FKB levels than non‐carriers, with female APP carriers having higher FKB levels than all non‐carriers.

**Conclusion:**

In a mouse cohort at a comparative human age of ∼70 years, lower brain SUVR in APP carriers coupled with lower FBG indicates bioenergetic deficits and the inability to meet energetic demands via glycolysis. This bioenergetic profile is also observed in clinical populations. Greater FKB in APP carriers suggests a shift toward utilizing fatty acids to meet bioenergetic demands. These results highlight the downstream metabolic impact of hAPP in the presence of hAPOE in these aged mice, providing important evidence for the interplay between AD genetic risk factors and bioenergetic dysfunction.

